# Antifeedant activity of invasive *Prunus serotina* leaves methanolic extract against *Sitophilus granarius*, a pest of stored products

**DOI:** 10.1038/s41598-025-10326-1

**Published:** 2025-07-15

**Authors:** Paulina Bączek, Jacek Łyczko, Kamila Twardowska, Mariusz Iwanowski, Iwona Gruss, Jacek Twardowski

**Affiliations:** 1 Department of Plant Protection, Wroclaw University of Environmental and Life Sciences, Wrocław, Poland; 2 Department of Food Chemistry and Biocatalysis, Wrocław University of Environmental and Life Sciences, Wrocław, Poland

**Keywords:** Plant-derived insecticide, Invasive plant species, Deterrent feeding activity, Storage pest control, Chemical ecology, Natural products

## Abstract

Synthetic pesticides pose a risk to the environment and human health by contaminating soil, water, and food chains. Natural plant-based alternatives offer a safer and more sustainable solution by reducing pollution, supporting biodiversity, and minimising pesticide resistance. This study evaluated the antifeedant activity of methanolic leaves extracts from invasive black cherry (*Prunus serotina* Erhr.) against a storage pest, the granary weevil (*Sitophilus granarius* L.). Chromatographic analysis of *P. serotina* leaves methanolic extracts identified 10 main phenolic compounds, with ursolic acid, p-coumaric acid o-coumaric acid, and caffeic acid exceeding 10%. LC-MS/MS analysis detected 12 compounds above the limit of quantification (LOQ), with luteolin-7-O-glucoside, caffeic acid, and chlorogenic acid at the highest concentrations. The antifeedant activity of *P. serotina* leaves methanolic extract was tested using the wheat wafer method, showing medium antifeedant effects at all extract concentrations (3.5, 5.0, and 12.0 mg/mL). Both males and females fed significantly less extract-treated wafers, with the inhibition of female feeding being stronger at 12.0 mg/ml. The extracts of *P. serotina* effectively discourage feeding of *S. granarius*, and the potency increases with concentration. Their flavonoids, phenolic acids, and cyanogenic glycosides suggest a complex mode of action, making them a promising natural alternative to synthetic insecticides. Further research should isolate key active compounds and evaluate their efficacy as botanical pesticides.

**Supplementary Information:**

The online version contains supplementary material available at 10.1038/s41598-025-10326-1.

## Introduction

Synthetic pesticides pose significant environmental and health risks in Europe, despite the introduction of new regulations. These chemicals contaminate soil, water, and food chains, affecting biodiversity and human health^1,2^. Numerous studies show that pesticides have led to declines in insect biomass, farmland birds, and pollinator populations^1^. On the other hand, insect pests are significant threat to stored food products, causing substantial losses and compromising food quality and safety^3,4^. These pests can reduce nutritional value, contaminate food with body fragments, and create unhygienic conditions^3^. Various control methods are used, including physical, chemical, and biological approaches^5^. Traditional pest management strategies involve synthetic pesticides, but concerns about pest resistance and environmental hazards have led to the exploration of alternatives such as biopesticides and other non-chemical methods^6^.

Granary weevil (*Sitophilus granarius* L.) has been a significant storage pest in Europe since the Neolithic period, with evidence of its early introduction found in Greece and central Europe^7^. This flightless weevil, along with other pests such as the Indian meal moth, continues to be a major problem in modern grain storage facilities, while mites, psocids, and other beetles are common secondary pests^8^.

Control of the grain weevil in stored cereals can be achieved through chemical and non-chemical methods. Chemical approaches include essential oils, spinetoram, and malathion (no longer approved for use in Europe), with malathion being the most effective^9^. The fatty acid composition of wheat kernels influences the development of *S. granarius*, and certain fatty acids can potentially stimulate or inhibit pest reproduction^10^. Varietal resistance is another non-chemical approach, as different wheat and corn genotypes exhibit varying levels of susceptibility to weevil attack^11^. Non-chemical methods for pest control in agriculture and storage have gained attention due to concerns about insecticide resistance, worker safety, and consumer demands for residue-free products^12^. More specifically, the management of *S. granarius* in stored grain involves not only conventional chemical compounds but also naturally derived phytochemicals. Susceptibility to infestation is further affected by phenolic and lipophilic compounds present in the grain, with higher levels of total lipids and sterols associated with increased vulnerability^13^. In search of alternatives to synthetic pesticides, plant extracts from *Achillea phrygia*, *Prangos ferulacea*, and *Salvia wiedemannii* have demonstrated both insecticidal and repellent properties against *S. granaries*^14^. In this context, bioactive phytochemicals are being actively investigated as eco-friendly and cost-effective solutions for pest management in stored grain systems^15^. Additionally, volatile compounds naturally emitted by *S. granarius*, such as 3-hydroxy-2-butanone and 1-pentadecene, have been found to trigger both electrophysiological and behavioral responses in the insects. These volatiles hold promise for the development of novel monitoring and control strategies, with 3-hydroxy-2-butanone functioning as an attractant at low concentrations and as a repellent at higher concentrations^16^. In Poland, the following substances are approved for the chemical control of the grain weevil: deltamethrin, pirimiphos-methyl, aluminum phosphide, and pyrethrins (https://www.gov.pl/web/rolnictwo/wyszukiwarka-srodkow-ochrony-roslin---zastosowanie).

Plant-derived insecticides can be a solution to replace chemical agents in pest control. Some invasive plant species (e.g. species of the *Solidago* or *Reynoutria* genus) are suspected to be a source of natural pesticides^17–20^. Black cherry (*Prunus serotina* Erhr.) is native to North America and was successfully introduced to Europe in the 17th century^21^. Issues related to its spread are studied in several countries, including Italy, Hungary, Belgium, Germany, the Netherlands, France, and Norway^22–28^. This alien species thrives in various habitats, demonstrating a strong capacity for both generative reproduction and vegetative sprouting. Its numerous seeds are widely dispersed by birds and mammals, and it establishes a long-lasting, shade-tolerant seedling bank^21,29,30^. It is suspected that the black cherry effectively competes with co-occurring species by producing and releasing allelochemicals^31–33^. *P. serotina* possesses strong constitutive chemical defences against herbivores, with key compounds including cyanogenic glycosides^34^ and phenolic compounds, primarily flavonoids^35^. Furthermore, amygdalin and prunasin (cyanogenic glycosides present in the leaves of *P. serotina*) have been shown to impede the feeding activities of the cherry-oat aphid, *Rhopalosiphum padi*^36^. The mechanism of the toxicity is the inhibition of cellular respiration by the release of toxic hydrogen cyanide (HCN) from cyanogenic glycosides^37^. Cyanogenesis is widely recognised as an effective herbivore defence mechanism^38,39^. Research has shown that within the context of cyanogenic plants, there is a trade-off between defence mechanisms against herbivores and pathogens^38^. In black cherry populations established in Europe, there has been a change in the concentration of cyanogenic glycosides compared to their native range, resulting in alterations in their resistance to leaf-eating insects^40^. A significant compound in *P. serotina* leaves are tannins, that can be categorised into two types: hydrolyzable tannins and non-hydrolyzable (or condensed) tannins. The function of hydrolyzable tannins is primarily to serve as a defence mechanism against herbivores^41,42^while condensed tannins primarily protect plants from pathogens^43^as demonstrated in *P. serotina*^44^. Consequently, black cherry extracts exhibit high biological activity. However, the insecticidal properties of black cherry leaf extracts have not previously been studied.

The purpose of the study was to determine the chemical composition of the methanolic extract of black cherry leaves and to evaluate its antifeeding activity against grain weevil.

The following research hypotheses have been formulated:

- Granary weevil feeding will be significantly reduced by methanolic extracts of black cherry leaves.
- The extent of feeding inhibition will depend on the concentration of the extract used.
- The effect of *P. serotina* leaf extracts on granary weevil feeding behaviour will not depend on sex.

## Materials and methods

### Collection of plant material

Material for the preparation of extracts: fresh black cherry leaves were collected from a fallow field in the city of Wrocław, Poland (51.168479 N, 17.009228 E). The leaves of *P. serotina* were collected at the beginning of leafing, in April 2024. The collected material was dried in the dark at a maximum temperature of 50 °C. After a constant dry mass, the leaves were powdered, and methanolic extracts were prepared from them.

### Extracts of black cherry leaves and preparation of samples for chromatographic analyses

All chemicals and reagents were purchased from Sigma-Aldrich (Steinheim, Germany). All extractions and analyses were performed in triplicate. The black cherry leaves extract method of^45^ with slight modifications was used. Briefly, 20 ± 0.05 g of dried and powdered black cherry leaves were weighed in a 250 mL volume glass flask and macerated for 24 h (120 rpm) with 200 mL of analytical grade 80% methanol. After the extraction process, the samples were centrifuged (30 min, 5000 rpm), filtered, and evaporated to dryness. The dried extract was weighted with an analytical scale. At this step, the extract was divided for insecticidal tests and further chemical analyses.

The extraction yield (per 100 g of dried material) was calculated according to equation:

$$\:\text{E}\text{x}\text{t}\text{r}\text{a}\text{c}\text{t}\text{i}\text{o}\text{n}\:\text{y}\text{i}\text{e}\text{l}\text{d}\:\left[\frac{\text{g}}{100\text{g}}\right]=\frac{\text{m}\text{a}\text{s}\text{s}\:\text{o}\text{f}\:\text{t}\text{h}\text{e}\:\text{d}\text{r}\text{i}\text{e}\text{d}\:\text{e}\text{x}\text{t}\text{r}\text{a}\text{c}\text{t}\:\left[\text{g}\right]\times\:100\:\left[\text{g}\right]}{20\:\left[\text{g}\right]}$$

For the GC-MS profiling, 10 mg of extract was weighted and suspended with 500 µL of pyridine and 50 µL of BSTFA for derivatization of the analytes was added. The silylation process was carried out for 45 min at 60 °C. Before the analysis, the sample was diluted 10 times with methyl tert-butyl ether. For LC-MS/MS analysis 10 mg of extract was suspended with 10 mL of pure, chromatographical grade methanol. For LC-MS/MS samples, they were diluted 1000 times (for hyperoside and chlorogenic acid analysis) and 100 times (the rest of the analytes).

### Total phenolic content (TPC) and total flavonoid content (TPC) of black cherry leaves methanolic extract

TPC and TFC were determined by the colorimetric method based on the^46^ method. Briefly, for the determination of TPC, 125 mL of black cherry methanolic solution (1 mg/mL) was mixed with 500 mL of distilled water and 125 mL of Folin–Ciocalteu reagent and kept for 6 min. Then, 1.25 mL of 7% sodium carbonate was added, and volume was set up for 3 mL in total with distilled water. After incubation for 90 min at room temperature, the absorbance was measured at 760 nm. Results were expressed as gallic acid equivalents (g GAE/100 g dw).

For TFC measurement 250 mL of black cherry methanolic solution (1 mg/mL) was mixed with 75 mL of sodium nitrate (5% solution) and kept for 6 min. Then 150 mL of aluminium(III) chloride solution (10% solution) and 500 mL of sodium hydroxide (1 M) were added. The final volume was set up for 2.5 mL with distilled water, the sample was vigorously shaken and incubated for 30 min at room temperature. The absorbance was measured at 517 nm and the results were expressed as quercetin equivalents (g QCE/100 g dw).

### GC-MS profiling of black cherry leaves methanolic extract

The GC-MS profiling of black cherry leaves extracts was carried out with Shimadzu GCMS QP 2020 (Shimadzu, Kyoto, Japan) equipped with SH-Rxi-5Sil MS (Shimadzu, Kyoto, Japan) column with dimensions 30 m × 0.25 mm × 0.25 μm phase thickness. 1 µL of the sample was injected at 280 °C with a split of 20. Helium with a linear velocity of 37.5 cm/s and a column flow of 1 mL/min was used as the carrier gas. Used for analytes, the separation program started at 100 °C held for 1 min and then raised to 190 °C at a rate of 2 °C/min, then to 300 at a rate of 5 °C/min and held for 25 min. The interface temperature was 270 °C and ion source temperature was 250 °C. Electron impact (EI) mode was used for analytic ionization at 70 eV. For analysis SCAN mode in the range 40–1000 *m/z* was used.

The compound’s identification was based on the comparison of experimentally obtained mass spectra with those available in NIST 20 (National Institute of Standards and Technology) library and literature^47,48^supported with pure analytical standards reference.

### LC-MS/MS analysis of black cherry leaves methanolic extract

The LC-MS/MS analysis was performed with LCMS-8045 (Shimadzu, Kyoto, Japan) equipped with AccucoreTM RP-MS column (Thermo Fisher Scientific, Waltham, MA, USA) with dimensions 2.6u, 100 A, 150 × 3.0 mm. As eluents 0.1% aqueous solution of formic acid (A) and 0.1% ACN solution of formic acid (B) were used. The analysis was performed with gradient program: start with 10% B, then 20% B in 5 min, then 60% B in 10 min, then 10% B in 13 min kept up to 17 min. The column flow was 0.35 mL/min and column oven temperature 45 °C.

The analysis of compounds was performed in MRM mode, which details are given in Table S1 (Supplementary) while the quantification was based on an external standard method, namely 5-points calibration curves. The selection of quantification analytes was based on the earlier research focus on growth in Poland *P. serotina*^49^namely, 4-hydroxybenzoic acid, caffeic acid, chlorogenic acid, ferulic acid, hyperoside, kempferol-3-rutinoside, luteolin-7-glucoside, o-coumaric acid, p-coumaric acid, quercetin-3-glucoside, quercetin, rutin and ursolic acid.

### Test of antifeedant activity of black cherry leaves methanolic extract

The feeding deterrent activity were carried out using granary weevil (*S. granarius*) species of stored product pest. *S. granarius* had been one of the stored pests selected originally by Nawrot et al.^50^ for its stored product pest status, and is still considered as model organisms for screening the antifeedant activity of chemical compounds^51–55^. The insects were reared in permanent darkness in climatic chambers at 24 ± 1 °C and relative humidity at 70 ± 5%. Granary weevil was offered wheat grain of cv. Natula as a substrate for food and oviposition. The tests were carried out in the same rearing chambers. The test insects were separated from the culture and food 24 h before the start of the tests. Adult grain weevils (at least 14 days old) were differentiated into males and females using differences in morphological characteristics (sexual dimorphism)^56^.

The ‘wheat wafer test’ is commonly used to evaluate the feeding deterrent activity against various insect pests^57–60^. It was run as described by Nawrot et al.^50,51^and identically as used more recently by Jackowski et al.^55,61^. Three concentrations of black cherry leaves methanolic extract were prepared and used for biotests: 3.5 mg/mL, 5.0 mg/mL and 12.0 mg/mL. To prepare individual concentrations, 99.9% ethanol (as a solvent) and a drop of tween 80 (as an emulsifier) were used. The methodology of the ‘wheat wafer test” is described in detail in Supplementary material.

### Statistical analysis

The total deterrency coefficient (T) and the loss of mass of wheat wafers (calculated in multiple choice tests - Supplementary) were used as an indicator of the biological activity of the extracts tested. Data sets for individual concentration of extract and sex of tested insects were checked for normality based on the Shapiro–Wilk W test. It turned out that the data did not have a normal distribution, so the nonparametric methods were used. Kruskal–Wallis analysis of the variance (ANOVA) of ranks was used to compare the T values obtained in wafer tests and feeding inhibition for a particular concentration of black cherry leaves methanolic extracts and insects sex. The Mann–Whitney U test was used for pairwise comparisons - wheat wafer mass loss for wheat wafer immersed in an extract of a given concentration versus wheat wafer immersed only in solvent (99.9% ethanol with one drop of tween 80). Significance was evaluated at *p* ≤ 0.05. The analyses were performed using STATISTICA software v. 13 (TIBCO Software Inc., Palo Alto, CA, USA).

## Results

### Chemical profile of black cherry leaves methanolic extract

The total yield of the methanolic extract of black cherry leaves used in this study was 17.16 ± 0.03 g per 100 g of dried leaves. The TPC in the crude extract was determined to be 2.440 g GAE per 100 g dry weight (dw), while the TFC reached 0.932 g QCE per 100 g dw.

As a first attempt, the main phenolic constituents of black cherry leaves were identified by the GC-MS technique, followed by derivatization of the analytes. This attempt allowed us to find 10 compounds (Table 1), while 9 were successfully identified. Among the compounds found, the derivative ursolic acid (31.34 ± 0.34%), p-coumaric acid derivative (21.18 ± 0.32%), o-coumaric acid derivative (11.34 ± 0.12%) and caffeic acid derivative (13.72 ± 0.09%) were found in amount greater than 10%.

**Table 1:** GC-MS profiling of black cherry leaves methanolic extract.

Compound	Mass spectra similarity [%]^1^	Share [%]^2^
Benzoic Acid, TMS derivative	95	5.69 ± 0.21
Mandelic acid, 2TMS derivative	94	2.64 ± 0.03
3-Phenyllactic acid, 2TMS derivative	96	3.12 ± 0.07
unknown phenolic acid TMS derivative		3.01 ± 0.05
*o*-Coumaric acid, 2TMS derivative	90	11.34 ± 0.12
*p*-Coumaric acid, 2TMS derivative	90	21.18 ± 0.32
Ferulic acid, 2TMS derivative	92	2.81 ± 0.14
Caffeic acid, 3TMS derivative	95	13.72 ± 0.09
Oleanolic acid 2TMS	90	5.15 ± 0.11
Ursolic acid 2TMS	92	31.34 ± 0.34

^1^Based on the comparison with NIST20 library; ^2^Calculated on the base of analytes peak area.

Among analysed by LC-MS/MS method 12 compounds were found with an amount higher than determined LOQ, while one was only with an amount higher than LOD (Table 2). The highest concentration was found for luteolin-7-*O*-glucoside, caffeic acid and chlorogenic acid, 273.43 ± 3.79, 215.86 ± 6.11 and 214.78 ± 6.62 µg/100 mg of extract, respectively, while the lowest amounts were found for ursolic acid, ferulic acid and *o*-coumaric acid, 17.14 ± 0.17, 29.69 ± 0.15 and 49.41 ± 1.50, respectively (Table 2).

**Table 2:** LC-MS/MS analysis of methanolic extract from *Prunus serotina* leaves.

Compound	µg/100 mg of extract	LOD [ng/ml]	LOQ [ng/ml]
4-hydroxybenzoic acid	tr	19.0	62.7
caffeic acid	215.86 ± 6.11	16.3	53.8
chlorogenic acid	214.78 ± 6.62	23.4	77.2
ferulic acid	29.69 ± 0.15	84.1	277.5
hyperoside	124.17 ± 4.70	39.9	131.7
kempferol-3-O-rutinoside	140.45 ± 3.62	51.2	169.0
luteolin-7-*O*-glucoside	273.43 ± 3.79	47.4	156.4
*o*-coumaric acid	49.41 ± 1.50	32.5	107.2
*p*-coumaric acid	123.35 ± 3.92	33.1	109.2
quercetin-3-O-glucoside	tr	43.7	144.2
quercetin	53.55 ± 1.18	47.0	155.1
rutin	171.55 ± 3.23	38.8	128.04
ursolic acid	17.14 ± 0.17	68.8	227.04

tr - below LOQ.

### Antifeedant activity of tested extract

The antifeedant activity of the methanolic extracts of the black cherry leaves was determined based on the calculation of three coefficients: relative (R), absolute (A), and total (T) deterrency (Table 3). The activity of three concentrations of the extract (3.5, 5.0 and 12.0 mg/mL) was tested against female and male granary weevil. Each concentration of the extract showed medium deterrent activity (T values between 51 and 100) (Table 3). Statistical analyses did not show significant differences in T coefficients between females and males (Table 3) and individual extract concentrations (Table S2 (Supplementary)).

**Table 3:** Average values ​​of indicators: relative (R), absolute (A) and total (T) deterrency coefficient depending on the concentration of the black cherry leaves methanolic extract and the sex of the tested pest. The differences in T coefficient values ​​between females and males for extracts of given concentrations were determined using Mann–Whitney U test (Z and p in the right-hand columns).

Extract concentration mg/mL	R		A		T				Antifeedant activity
	Female	Male	Female	Male	Female	Male	Z	*p*	
12.0	96.4	71.4	−10.6	7.5	85.8	78.9	−1.4623	0.14367	Medium
5.0	68.5	67.4	−5	1.2	63.6	68.6	−0.4178	0.6761	Medium
3.5	60.8	72.9	3.1	2.3	63.9	75.3	−0.8356	0.4034	Medium

**Fig. 1:**
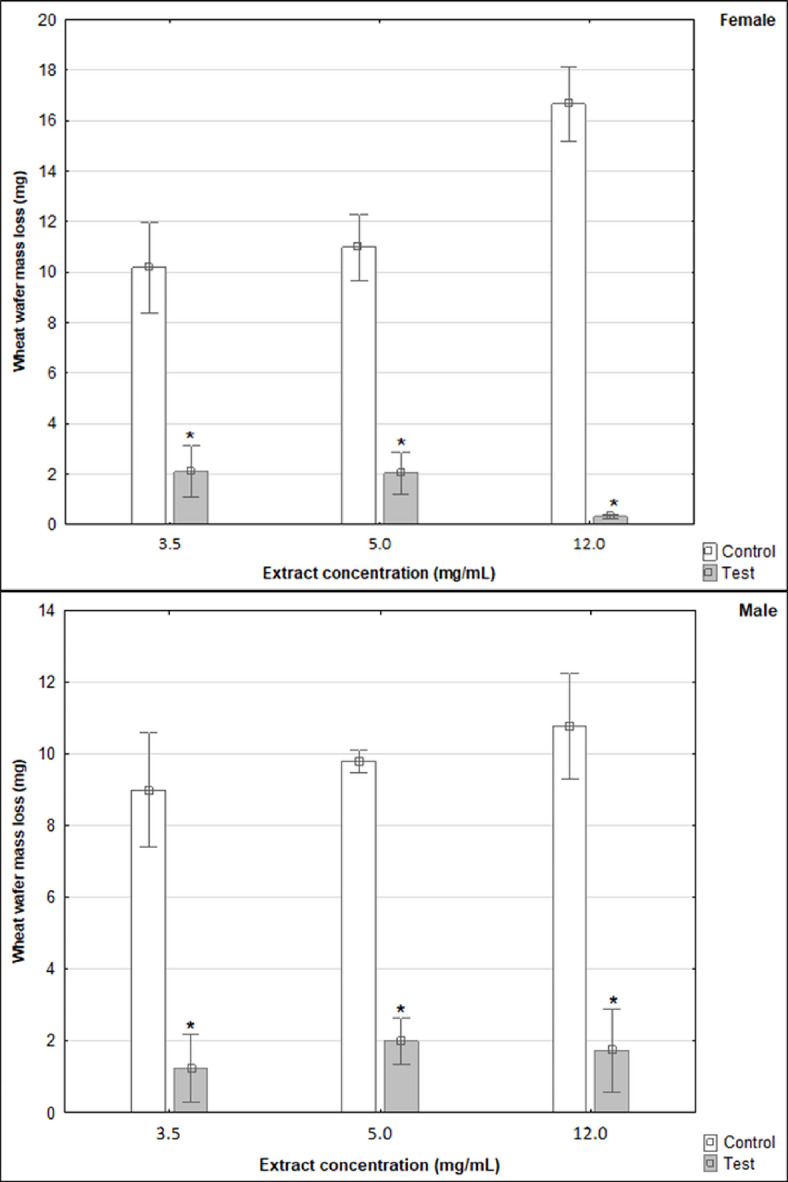
Loss of wheat wafer mass as a result of feeding by *Sitophilus granarius* females and males under the influence of black cherry leaves methanolic extract of various concentrations (Mean ± SE). Significant differences between pairs (control vs. tested concentration of the *Prunus serotina* extract) were estimated using the Mann–Whitney U test, marked with *n = 5.

Further statistical analysis was performed based on the wheat wafers mass loss from choice test. For each concentration of the methanolic extract from the leaves of black cherry, the mass loss of the reference wheat wafer was compared with the mass loss of the extract-treated wafers. Regardless of the concentration of the extract used, the female grain weevils fed significantly more on the control wheat wafers than on the wheat wafers treated with black cherry leaf extract (Table S3 (Supplementary), Fig. 1). Furthermore, significant inhibition of feeding (loss of weight of the wheat wafer) was also observed in males (Table S3 (Supplementary), Fig. 1). In more detail, the loss of mass of the wheat wafer was significantly lower compared to the control using all doses of extract (3.5, 5.0 and 12.0 mg/mL) (Table S3 (Supplementary), Fig. 1).

**Fig. 2:**
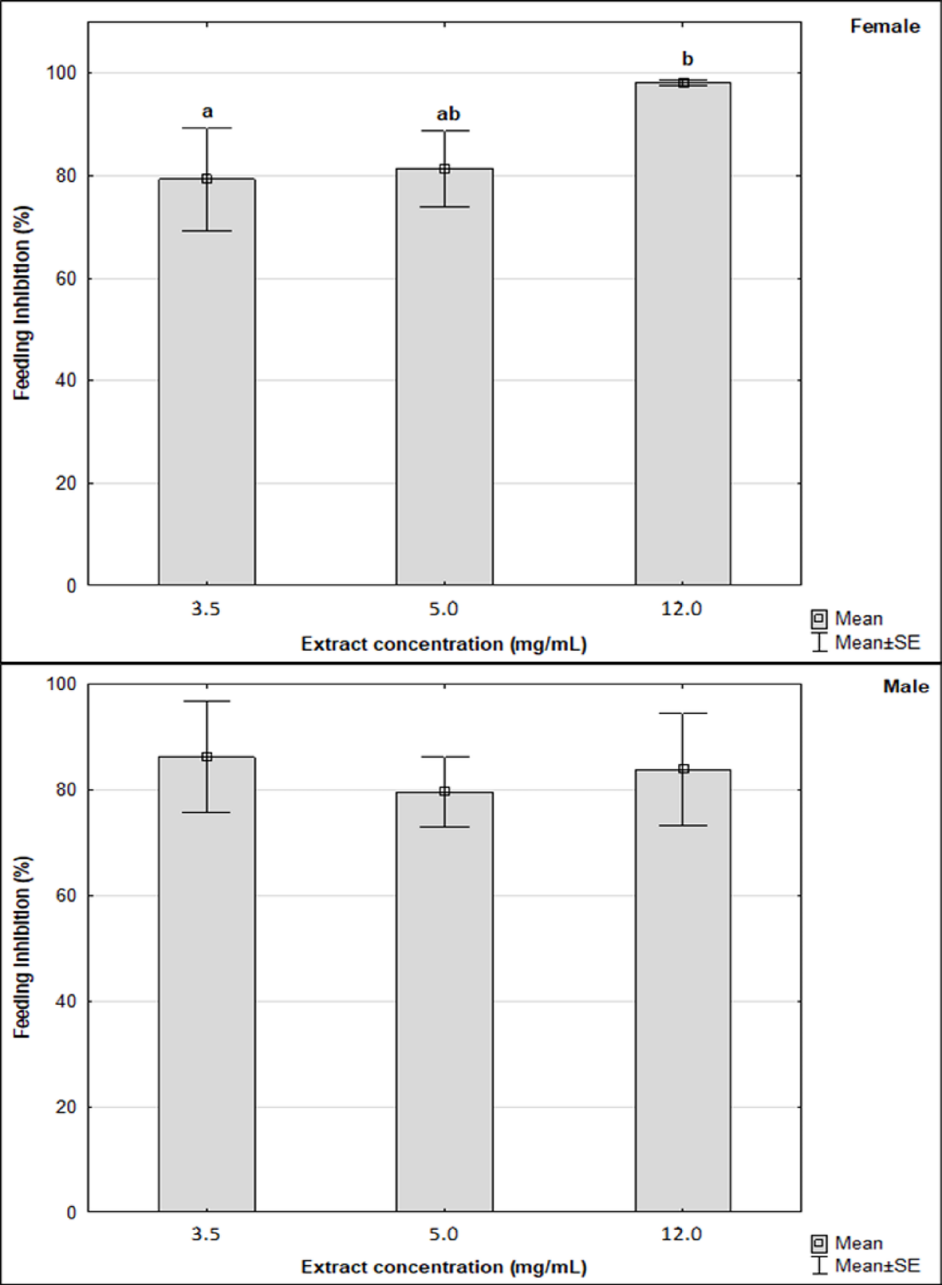
Feeding inhibition of female and male grain weevils under the influence of black cherry leaves methanolic extract of various concentrations (Mean ± SE). Significant differences were estimated using the Kruskal–Wallis analysis of variance (ANOVA), marked with letters, *n* = 5.

Furthermore, feeding inhibition of *S. granarius* female was found to be significantly higher when using the *P. serotina* leaf extract at a concentration of 12.0 mg/mL compared to inhibition under the influence of the extract at a concentration of 3.5 mg/mL (H = 6.86; *p* = 0.0324) (Fig. 2). Inhibition of feeding of male grain weevils did not differ significantly between the concentrations of the extract of black cherry leaves used (H = 2.06; *p* = 0.3564) (Fig. 2). Thus, the effects were sex-dependent.

## Discussion

*Prunus serotina*, commonly known as black cherry, exhibits significant biological activity due to its diverse phytochemical composition. Research has identified bioactive compounds such as polyphenols, anthocyanins, and flavonoids, which contribute to their strong antioxidant, antimicrobial, and pharmacological properties^49,62^. Our research has shown that TPC (total phenolic content) and TFC (total flavonoid content) in the crude extract were 2.440 g GAE (gallic acid equivalent)/100 g dw and 0.932 g QCE (quercetin equivalent)/100 g dw, respectively. In terms of chemical composition, the findings of the present study are consistent with previous studies on black cherry samples collected in Poland. For example^49^, reported a comparable TPC content of 2.154 g GAE per 100 g dw. However^63^, identified significantly higher levels of TPC, 3.27–5.11% dw. These variations can be attributed to different environmental factors during black cherry growth, including soil quality, temperature, humidity, and other conditions.

GC-MS analysis identified 9 of 10 phenolic compounds, with ursolic acid, p-coumaric acid, o-coumaric acid, and caffeic acid (13.72%) being the most abundant. LC-MS/MS detected 12 compounds above the LOQ, with luteolin-7-*O*-glucoside, caffeic acid, and chlorogenic acid at the highest concentrations, while ursolic acid, ferulic acid, and *o*-coumaric acid had the lowest. As may be observed, there were significant differences between quantitative and qualitative analyses between the results of the GC-MS and LC-MS/MS techniques. The GC-MS technique did not allow identifying flavonoid compounds, which was expected; however, the oleanoic acid and ursolic acid was surprising, which was not observed by LC-MS/MS analysis. The reason for this may be found with the sample preparation procedure (derivatization for GC-MS) and the basic principles of the techniques which were shown also in earlier studies^45,64^. Regarding the nonvolatile compounds profile, presented in this study results show some differences in comparison to other studies such as^35,49^or Olszewska and Kwapisz (2011), however, the reasons of that may be found with different techniques of extraction, different plant material, or different plant parts.

Despite extensive studies on its medicinal and antioxidant potential, the insecticidal properties of *P. serotina* remain largely unexplored. The ethanolic extracts of *P. serotina* fruit have shown antimicrobial activity against gram-negative bacteria and *Staphylococcus aureus*^65^while its bark extract has shown cysticidal effects against *Taenia crassiceps* (tapeworm), with naringenin identified as a key active compound^66^. The leaves contain vasorelaxant constituents, such as hyperoside, prunin, and ursolic acid, that induce vascular smooth muscle relaxation^67^. Comparative studies indicate that *P. padus* leaves exhibit higher antioxidant and antimicrobial activities than *P. serotina*^49^.

The insecticidal potential of other *Prunus* species has been partially explored. For example, methanolic extracts of *Prunus armeniaca* (apricot) kernels exhibit significant toxicity against *Tribolium confusum*^68^while *P. persica* extracts show limited insecticidal activity^69^. This study demonstrates that *P. serotina* methanolic extracts effectively reduce the feeding activity of male and female *S. granarius*. The antifeedant effect increases with the concentration of the extract, ranging from 3.5 to 12.0 mg/ml. For comparison, the concentration of deltamethrin used to protect the grain against *S. granarius* is 25 mg/mL in solution (Plan protection procucts in Poland). The repellent activity was also more distinct for the females compared to the males of *S. granarius*. According to other studies, various plant extracts, including those from *Achillea wilhelmsii*, *Capsicum annuum*, and *Melaleuca alternifolia*, have also shown promising insecticidal effects against *S. granarius*^70^. Lichen extracts of *Lecanora muralis*, *Letharia vulpina*, and *Peltigera rufescens* also demonstrated high mortality rates against adult *S. granarius*, with increased effectiveness at higher concentrations and longer exposure times^71^. Furthermore, *Achillea phrygia*, *Prangos ferulacea*, and *Salvia wiedemannii* exhibited both insecticidal and repellent properties against *S. granarius*^14^. It was also found that the feeding and oviposition behaviour of *S. granarius* is influenced by various wheat extracts and environmental factors, and the olfactory sensilla plays a crucial role in detecting these stimuli (Levinson and Kanaujia, 1982).

LC-MS/MS analysis identified several bioactive compounds extracted from the leaves of *P. serotina*, including luteolin-7-*O*-glucoside, caffeic acid, and chlorogenic acid. Chlorogenic acid showed high toxicity against agricultural pests such as *Bemisia tabaci* and *Spodoptera frugiperda*^20,72^. Flavonoids such as quercetin and kaempferol, detected in small amounts, are known for their insecticidal and deterrent properties^73,74^. Cyanogenic glycosides, present in *P. serotina*, serve as chemical defences, releasing toxic hydrogen cyanide upon tissue damage^75^. Other studies indicate that both natural and synthetic cyanohydrins effectively act as fumigants against stored-product insects^76^. It is important to note, that the interactions between compounds in mixtures can lead to complex effects that differ from those of individual substances, not explored in this research^77^. Furthermore, the presence of minor constituents such as flavonoids and cyanogenic glycosides can modulate the action of primary compounds, contributing to the broader ecological role of plants as natural pest control agents^78^.

## Conclusions

The methanolic extracts of *P. serotina* effectively discourage the feeding of *S. granarius*, the potency increasing alongside the concentration of the extract. The presence of flavonoids, phenolic acids, and cyanogenic glycosides suggests a multifaceted mode of action, potentially making *P. serotina* extracts a viable and environmentally friendly alternative to synthetic insecticides. Future studies should focus on isolating specific compounds responsible for the insecticidal effect and comparing their efficacy against other plant-based pesticides.

## Electronic supplementary material

Below is the link to the electronic supplementary material.

Supplementary Material 1

## Data Availability

The datasets generated during and analysed during the current study are available from the corresponding author on reasonable request.
