# Supplementary material for: Antifeedant activity of invasive Prunus serotina leaves methanolic extract against Sitophilus granarius, a pest of stored products
Source: Sci Rep. 2025 Jul 15;15:25469. doi: 10.1038/s41598-025-10326-1 (PMC12264154; doi:10.1038/s41598-025-10326-1)
Supplement: Supplementary file 1 — Supplementary Material 1 [file 41598_2025_10326_MOESM1_ESM.pdf]

Title: Antifeedant activity of invasive *Prunus serotina* leaves methanolic extract against *Sitophilus granarius*, a pest of stored products

Authors: Paulina Bączek[1], Jacek Łyczko[2], Kamila Twardowska[1], Mariusz Iwanowski[2], Iwona Gruss[1], Jacek Twardowski[1]

Authors' institutional affiliations:

1. Department of Plant Protection, Wroclaw University of Environmental and Life Sciences, Wrocław, Poland

2. Department of Food Chemistry and Biocatalysis, Wrocław University of Environmental and Life Sciences, Wrocław, Poland

Scientific Reports

Supplementary

1. Tables
2. Methodology of 'Wheat wafer test'

1. Tables

Table S1 LC-MS/MS's MRM analysis details.

**Compound**	**Ionization mode**	**Precursor ion [*m/z*]**	**Product ions [*m/z*]**
4-hydroxycynamic acid	negative	137.4	92.95
			65.05
caffeic acid	negative	179.4	135.05
			133.95
			107.0
chlorogenic acid	negative	353.0	191.3
			85.0
			93.05
ferulic acid	negative	193.4	134.0
			178.0
			149.2
hyperoside	negative	463.3	300.1
			271.15
			301.1
kempferol-3-rutinoside	negative	593.2	285.15
			284.15
			255.15
luteolin-7-glucoside	positive	449.2	287.0
			384.85
			417.05
o-coumaric acid	negative	163.2	119.1
p-coumaric acid			93.1
			117.1

quercetin-3-glucoside	negative	462.5	300.1<br>301.15
quercetin	negative	301.2	151.0<br>179.0<br>121.0
rutin	negative	609.3	300.15<br>301.1<br>271.25
ursolic acid	positive	457.5	411.25<br>439.15<br>95.0

Table S2 The results Kruskal–Wallis analysis of variance (ANOVA) showed no differences in T coefficient values between individual concentrations (3.5, 5.0, 12.0 mg/mL) of black cherry leaves methanolic extract.

	**H**	**p**
**Female**	3.5	0.17
**Male**	1.82	0.4

Table S3 The results of paired comparisons (the Mann–Whitney U test) - Influence of *Prunus serotina* leaves methanolic extract on loss of wheat wafer mass (control vs. tested concentration of the black cherry leaves methanolic extract) after 120 hours of *S. granarius* feeding. Significant differences marked *, n = 5.

**Extract concentration mg/mL**	**Female**		**Male**	
	**Z**	**p**	**Z**	**p**
3.5	-2.3	0.0*	-2.3	0.02*
5.0	-2.5	0.0*	-2.5	0.01*
12.0	-2.5	0.0*	-2.3	0.02*

2. Methodology of 'Wheat wafer test'

The 1.5 cm in diameter wheat wafers were cut out. These disks were immersed in a solution containing either the extract at a given concentration or only the solvent with one drop of tween 80 (for control/reference purposes) using forceps. The treated and reference disks are placed on separate large glass Petri dishes (210 mm diameter) and allowed to air-dry for 30 minutes. After drying, the disks are weighed. Then, they are transferred to smaller polystyrene Petri dishes (90 mm diameter). Test insects (adult female or male grain weevils) were introduced into the Petri dishes containing the wafers. The Petri dishes were then closed with lids and placed in controlled climatic conditions appropriate for the insects being tested. Each experiment was replicated five times. Five adult females or males of *S. granarius* were used for each replication. After 120 hours (5 days), the insects were removed from the Petri dishes. The remaining wheat wafer material was weighed again to assess the effect of treatment. The biotests were conducted in a three-part run simultaneously: reference (two wheat wafers treated with solvent alone), choice test (one solvent-treated wafer and one extract solution-treated wafer), and no-choice test (two extract solution-treated wafers). The depletion of wafer weight (which indicates how much was consumed) was used to calculate three deterrence coefficients: relative (R), absolute (A) and total (T) deterrence coefficient.

The relative deterrence coefficient (R) measures how much less (or more) the compound-treated wafer is consumed compared to the solvent-treated wafer in the choice test (where both options are presented).

Formula:

$$\mathrm{R} = \frac{\mathrm{C} - \mathrm{E}}{\mathrm{C} + \mathrm{E}} \times 100 \text{ (choice test)}$$

Where:
C = amount of reference (solvent-treated) wafer consumed in the choice test.
E = amount of compound-treated wafer consumed in the choice test.

The absolute deterrence coefficient (A) compares the consumption of wafers between the no-choice test (both wafers are extract-treated) and the reference (both wafers are solvent-treated).

Formula:

$$\mathrm{A} = \frac{\mathrm{CC} - \mathrm{EE}}{\mathrm{CC} + \mathrm{EE}} \times 100 \text{ (no} - \text{choice test)}$$

Where:
CC = amount of reference (solvent-treated) wafers consumed in the no-choice test.
EE = amount of extract-treated wafers consumed in the no-choice test

Total deterrence (T) is the sum of the relative and absolute deterrence coefficients.

Formula:

$$\mathrm{T} = \mathrm{A} + \mathrm{R}$$

The T coefficient is the key parameter used to assess the activity of a compound in terms of deterrence or promotion of feeding behaviour. Here is how to interpret the T values:

Very good deterrent Activity: T values between 151 and 200.
Good deterrent activity: T values between 101 and 150.

Medium deterrent activity: T values between 51 and 100.
Weak deterrent activity: T values below 50.

Negative T values indicate increased feeding rather than deterrence. The R and A values, which could be other experimental parameters, should not be used in isolation to assess whether the compound is a deterrent or a feeding promoter.

In addition, the weight loss of wheat wafers from the choice test was used to calculate the inhibition of grain weevil feeding. Feeding inhibition was calculated using the following formula:

$$\text{Feeding inhibition} = \frac{\text{weight loss of wheat wafer treated with extract } \times 100}{\text{weight loss of the control wheat wafer}} - 100$$
